# Buffalo Academy of Medicine

**Published:** 1893-01

**Authors:** 

**Affiliations:** Secretary


### Society Proceedings.

### BUFFALO ACADEMY OF MEDICINE.

*Stated Meeting held December 6, 1892.*

Reported and edited by WM. C. KRAUSS, M. D., Secretary.

C. C. FREDERICK, M. D., presiding.

Dr. MYNTER read a paper on Surgery of the Kidney, and Dr. PARK read a paper on Actinomycosis. (See Original Communications, pp. 325 and 338.)

### DISCUSSION.

Dr. H. E. HAYD: I am satisfied that the floating kidney exists more frequently than most of us are aware of, and it has been my experience that the diagnosis is very often made by the patients themselves. All reflex disturbances are apt to be overlooked, and particularly when the pathological condition produces so many and so varied symptomatology. Of late I am questioning more and more this so-called nervous dyspepsia, and in these cases always examine carefully for the floating kidney. If the patient be placed on her back, the kidney may escape detection, unless perchance found in the altered position, but by tilting the patient on the side and deep pressure made on the loin, this conical-shaped body may be very easily detected; in fact, the patient will often call your attention to this lump, which comes and goes, as she puts it. These cases of Dr. Mynter I saw and assisted him in the operation. One case was a particularly interesting one, since the patient was operated upon with the expectation of opening the gall-bladder and removing a handful of gall-stones. The woman had been seen by me in three definite attacks of gall-stone colic, great pain over the region of the gall-bladder, vomiting clay-colored stones, high colored urine and discolored conjunctivæ. The stools were carefully examined, but no gall-stones found. An operation was advised, and upon opening the abdomen, to our surprise we found a round mass pushing its way from behind the mesentery forward. Since the operation the patient has been very much worse. This mass is now fixed forward by adhesion, but can, no doubt, be released by a subsequent operation. I don't believe the results of nephrorraphy are as satisfactory as some of the statistics which Dr. Mynter gives us. Unfortunately, these women continue to suffer a great deal, perhaps, from the fixative adhesions, but as the operation is simple, easily done, and practically without danger, I feel that it is justifiable.

Dr. R. Park: I have operated upon the kidney many times. The first case was that of a child, twenty-three months old. I removed a fibro-cystic kidney, by abdominal section, and the child recovered. The other kidney became similarly affected soon thereafter, and the child died. The operation of nephrorraphy I have done twice. The first case was that of a young man, concerning whose affection there was a difference of opinion. I made a laparatomy, found a floating kidney, then made a lumbar section and fixed it. He recovered. After a time, he desired to have it removed, on account of the pain and other disagreeable symptoms. On removing it, I was struck by the density of the adhesions about it. The pain may have been due to these adhesions. He is fully recovered now. Last Winter, I had a case in the hospital of a woman with two floating kidneys. It was impossible to tell which was the more movable, so I made a laparatomy, decided upon the one which I thought was the more movable, then made a lumbar section and fixed it. Last Summer, I had a case of abscess of the kidney. I operated, and found a tubercular kidney, about as large as a pitcher. I removed all the cheesy material, closed the wound, and the woman recovered. Some pus comes from the kidney, but whether urine does or not, I cannot say.

Dr. H. Mynter: As to the frequency of floating kidney, I have seen many cases during the past few years. I think it is more frequent than we suppose. Where one kidney is healthy, I advise nephrectomy of the floating kidney. I do not regard the operation as a very serious one.

Dr. F. S. Metcalfe: Regarding the treatment of actinomycosis, I am informed that in Chicago, under the supervision of the Department of Agriculture, mild cases are treated successfully with the iodide of potash.

Dr. R. Park: Iodine has been injected hypodermically, in cases of actinomycosis, with but little success. Electrolysis has little power over these masses.

---

The wonderful progress made in surgery is shown from the fact that only nine per cent. of all operations in amputation are fatal.—*Omaha Clinic.*

